# Primary Squamous Cell Carcinoma of the Thyroid: A Case Report and Literature Review About a Rare Entity

**DOI:** 10.7759/cureus.14963

**Published:** 2021-05-11

**Authors:** Noha N Soror, Parth Shah, Lori Hemrock, Robert Bennett

**Affiliations:** 1 Internal Medicine, Western Reserve Health Education/NEOMED, Warren, USA; 2 Medical Oncology, The Hope Center for Cancer Care, Warren, USA; 3 Pathology, Steward Health Care, Warren, USA

**Keywords:** thyroid cancer, squamous cell carcinoma, squamous cell carcinoma (scc), thryroid

## Abstract

Primary squamous cell carcinoma of the thyroid (PSCCT) is a rare and aggressive neoplasm that accounts for less than 1% of all thyroid malignancies. Its incidence is higher in females in their fifth and sixth decades of life. The pathophysiology of PSCCT is still a subject of debate as squamous cells are generally absent in the thyroid gland under normal physiologic conditions. Immunohistochemistry is important for reaching a definite diagnosis as it helps to differentiate PSCCT from metastases from other primary sites. Clinically, PSCCT usually presents as a rapidly enlarging anterior neck mass. Surgical resection is the mainstay of treatment despite the lack of any standard guidelines for the same given the rarity of the disease. Adjuvant chemotherapy, radiotherapy, and targeted therapy continue to be of unclear benefit. We report a case of PSSCT in a male patient who presented with a rapidly enlarging neck mass.

## Introduction

Primary squamous cell carcinoma of the thyroid (PSCCT) is a rare tumor of the thyroid gland with a higher rate of incidence in females in their fifth and sixth decades of life [1]. The condition is associated with a very poor prognosis and has a five-year overall survival (OS) rate of only 17.7% [2]. It usually presents as a rapidly enlarging anterior neck mass. Other common symptoms include dyspnea, dysphagia, and voice changes [3]. The diagnosis of PSCCT is usually challenging, and ruling out metastasis from other primary sites is mandatory for a proper diagnosis; hence, immunohistochemistry is of crucial importance. There is very limited data in the literature to guide treatment and appropriate follow-up [4].

## Case presentation

**Figure 1:**
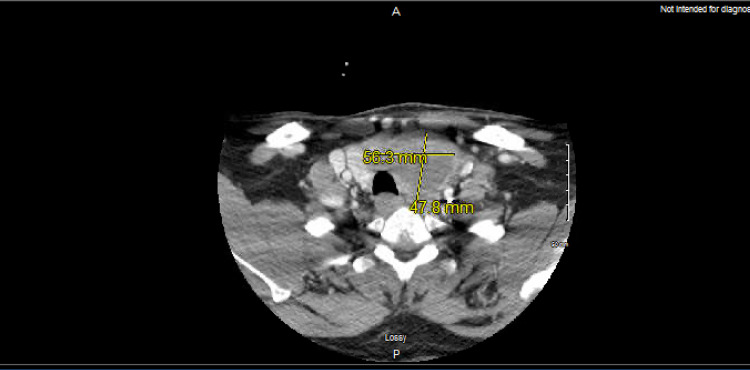
CT neck with contrast showing the thyroid mass CT: computed tomography

A 53-year-old white male with no significant past medical or family history presented with a painless rapidly enlarging left-sided neck mass for a period of over a month. Associated symptoms included difficulty in swallowing and weight loss. The patient was a lifetime non-smoker and had no history of alcohol abuse. An ultrasound of the neck was performed, which demonstrated an isoechoic nodule in the left thyroid lobe. Also, a CT scan with contrast of the neck was ordered, which showed a large hypo-dense heterogeneously enhancing mass involving the left lobe of the thyroid, extending across the isthmus towards the right lobe. Enlarged hypo-dense left supra-clavicular and left cervical lymph nodes were also noted (Figure 1).

Following these results, a needle aspiration biopsy of thyroid mass was performed, which demonstrated a poorly differentiated malignancy suggesting squamous cell carcinoma of the thyroid. The patient was admitted to the hospital for a total thyroidectomy with left modified radical neck dissection and left central node dissection.

**Figure 2:**
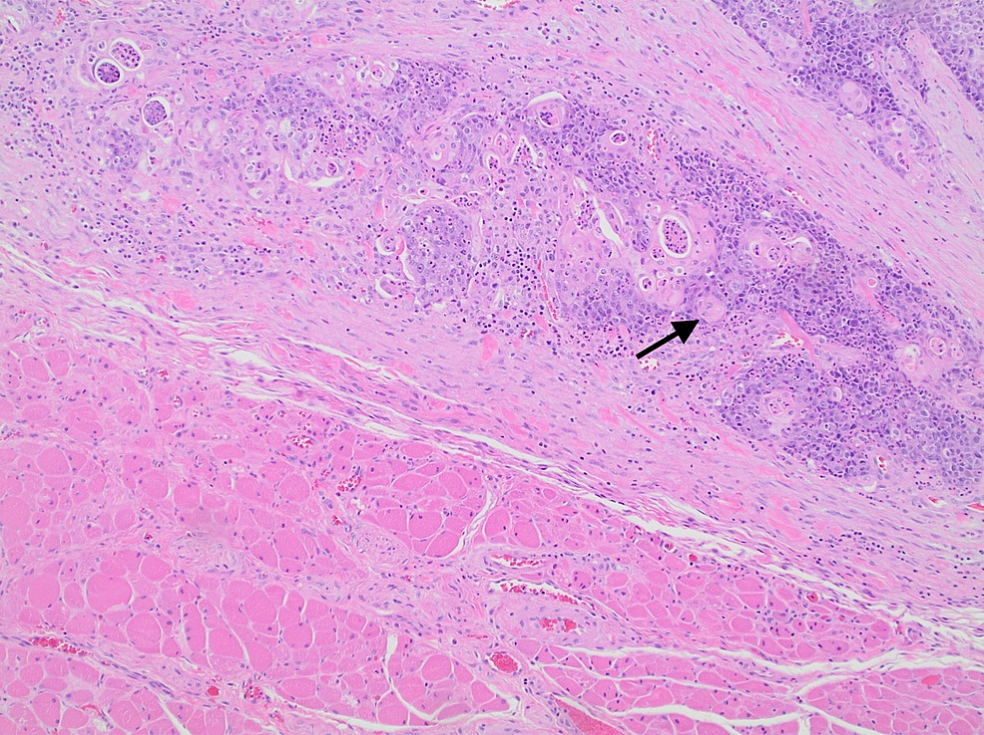
PSCCT invading parathyroid skeletal muscle (arrow) (10X) PSCCT: primary squamous cell carcinoma of the thyroid

**Figure 3:**
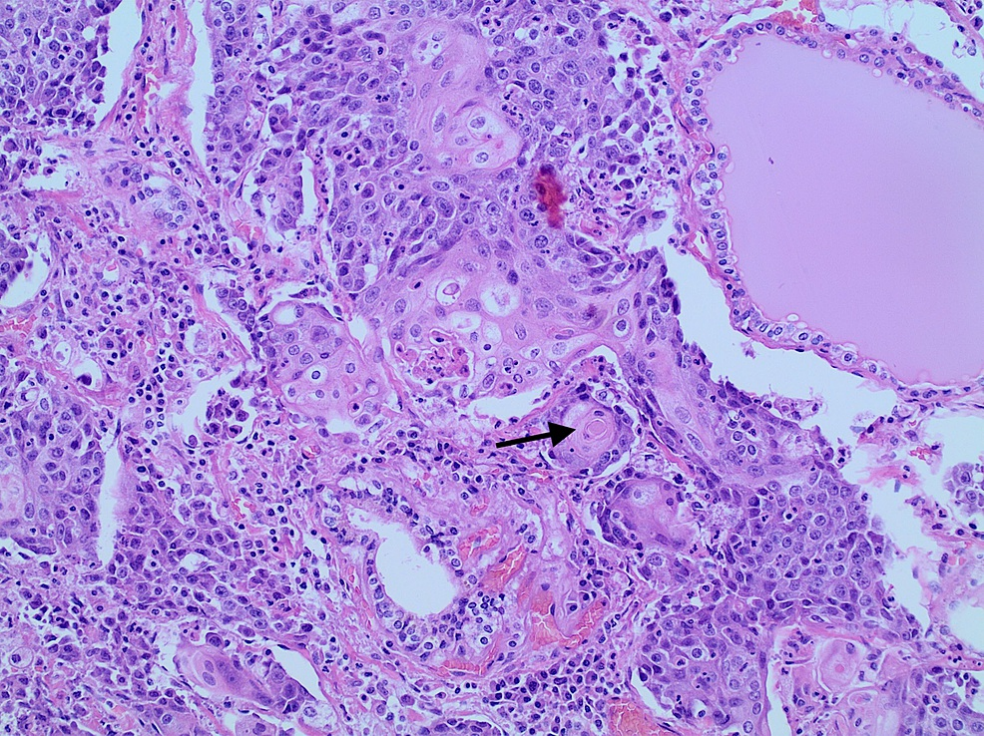
High magnification squamous cell carcinoma of the thyroid (arrow) (40X)

**Figure 4:**
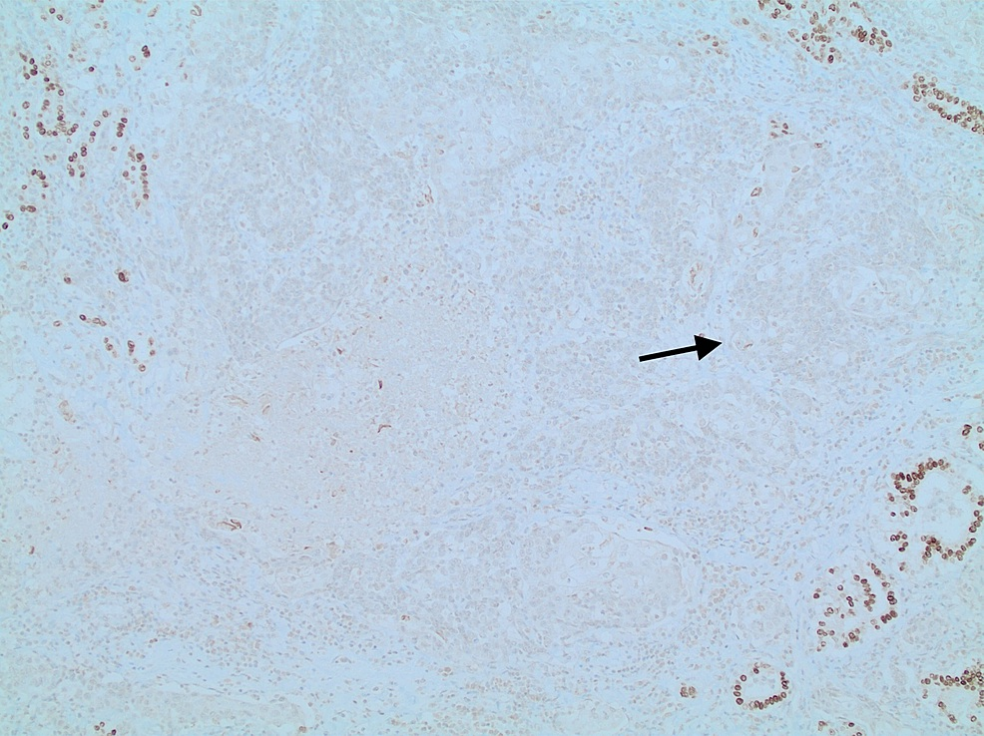
TTF1-negative tumor cells while surrounding benign thyroid follicles are positive (arrow) TTF1: thyroid transcription factor 1

**Figure 5:**
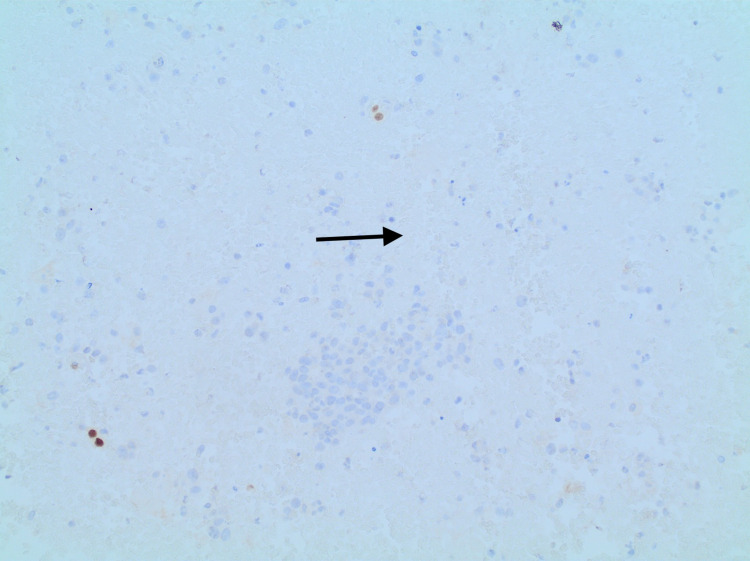
PAX8-negative tumor cells (arrow) PAX8: paired box gene 8

**Figure 6:**
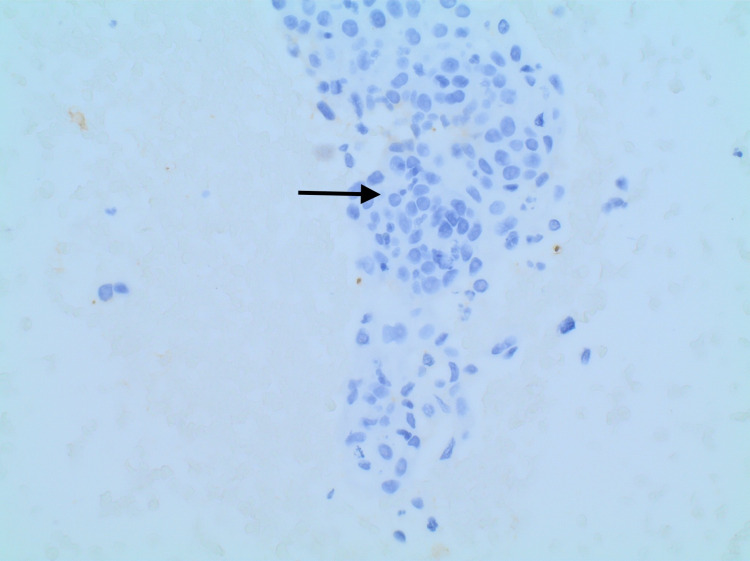
Thyroglobulin-negative tumor cells (arrow)

Pathology report of the tumor mass showed invasive, moderately differentiated squamous cell carcinoma (Figures 2, 3) with angiolymphatic and extrathyroidal extension involving the left lobe, right lobe, and isthmus; the enlargement of one of two anterior perithyroidal lymph nodes was also reported (pT3b, pN1b) (Figure 3). No definitive glandular differentiation was seen. Immunohistochemically, the neoplastic squamous cells were thyroid transcription factor 1 (TTF1)-negative (Figure 4), paired box gene 8 (PAX8)-negative (Figure 5), thyroglobulin-negative (Figure 6), and CD5-negative, suggesting that the tumor arose from a thyroid primary. Left cervical lymph node resection demonstrated one of six level II lymph nodes, three to four level III lymph nodes, and three of three level IV lymph nodes involved with the disease.

On the third postoperative day, the patient started complaining of shortness of breath for which a chest X-ray was obtained. The X-ray revealed bilateral pleural effusion, more on the left than the right side. Thoracentesis was performed; pleural fluid was exudative, but negative for malignant cells. Bone scan and CT abdomen/pelvis were both negative for metastasis. The patient was started on supplemental levothyroxine and was discharged home. Over the next two months, the patient presented again to the hospital with worsening shortness of breath. CT of the chest/abdomen/pelvis was obtained, which revealed a new L hilar mass, multifocal lung consolidations consistent with metastasis, as well as enlarged mediastinal lymph nodes. The patient was started on carboplatin/5-FU/pembrolizumab in two cycles with disease progression. A repeat CT chest after the second cycle revealed interval increased size of the left hilar mass, complex left pleural fluid with extensive pleural thickening, as well as multifocal left lung consolidation and mediastinal lymphadenopathy. Given the disease progression following first-line treatment, a discussion was conducted about choosing either second-line cetuximab versus clinical trial. However, the patient's worsening kidney function and performance status precluded such plans. The patient and his family refused dialysis and chose hospice care. Unfortunately, the patient passed away a few weeks later.

## Discussion

PSCCT is a rare but aggressive neoplasm that accounts for less than 1% of all thyroid malignancies [1]. Notably, its incidence is higher in females and it usually affects older patients in their fifth and sixth decades of life. The female-to-male ratio is 2.4:1 [2]. The condition has a very poor prognosis with a five-year overall survival (OS) of 17.7% [3]. In the literature, PSCCT is reported to be likely associated with other thyroid diseases such as Hashimoto's thyroiditis, papillary thyroid cancer (PTC), follicular carcinoma, and anaplastic carcinoma [4]. Of note, the annual incidence of PSCCT has decreased from 0.4% in 1973 to 0.1% in 2015. This drop could be explained by earlier diagnosis and treatment of related thyroid conditions as well as the advancement in immunophenotyping technology to reach a definite diagnosis. It is suggested that even the low number of cases reported currently seems to be overestimated. As per a study based on the American College of Surgeons National Cancer Database, 314 cases of PSCCT were diagnosed between 2004 and 2015 [5]. Geographically, almost half of the cases are reported from Asia, with Japan accounting for the most number of cases. The rest of the cases were reported from the USA and the UK in equal numbers (25%) [6].

PSCCT usually presents clinically as a rapidly enlarging anterior neck mass (60%); dyspnea, dysphagia, and voice changes are also common presenting symptoms [3]. Diagnosis is usually challenging as metastasis or adjacent organ tumor invasion has to be ruled out [7]. 

Squamous cells are absent in the thyroid gland under normal physiologic conditions and for this reason, the pathophysiology of PSCCT has been a subject of debate. Three theories have been proposed to explain the development of squamous cells within the thyroid gland, and the mechanisms these theories have put forward can be broadly summarized as follows: an embryonic origin involving branchial arch/thyroglossal duct remnants; metaplastic transformations within the thyroid gland likely secondary to chronic inflammatory process; or dedifferentiation of another existing primary thyroid cancer (e.g., anaplastic, papillary, or medullary carcinomas). The observation that up to 40% of PTCs and many anaplastic thyroid cancers contain patchy regions of the squamous cell population supports the dedifferentiation theory [8].

Immunohistochemistry is crucial for the diagnosis of PSCCT as it helps to differentiate primary thyroid tumors from metastases from other primary sites [9]. Positivity for CK5/6 and CK7 confirms a carcinoma to be of squamous cell origin [10], while negative TTF1 and thyroglobulin help to rule out other thyroid neoplasms including papillary and follicular thyroid carcinoma. A negative CD5 immunostaining militates against the diagnosis of carcinoma showing thymus-like differentiation (CASTLE). PSCCT rarely stains for thyroglobulin or TTF1 [11]. Most thyroid carcinomas express PAX8 transcription factor; however, the details about PAX8 expression in PSCCT currently remain unknown. Positive PAX8 is usually used to confirm a primary thyroid etiology, However, this likely happens in conjunction with other histological thyroid cancer subtypes. It has been reported that pure SCC of the thyroid lacks PAX8 expression [12].

Treatment-wise, there are limited data regarding outcomes given the rarity of the disease. It is almost exclusively reported as case reports and small case series in the literature; there is one analysis based on the Surveillance, Epidemiology, and End Results (SEER) program database, but unfortunately, it does not include complete treatment data [13]. Surgical resection is usually the recommended curative option. The extent of the surgical resection is poorly defined with a high failure rate. Advanced stage diseases at presentation and the invasive nature of the PSCCT are the main reasons for surgical failure [2]. PSCCT is relatively resistant to radiotherapy compared to other squamous cell carcinomas. Some reports have indicated that the complete eradication of the disease can be achieved in patients receiving both surgery and adjuvant radiation. The benefits of adjuvant chemotherapy and radiotherapy remain unclear so far [3]. Targeted therapy using receptor kinase inhibitors that inhibit different signaling pathways are also considered for the treatment of aggressive recurrent thyroid cancers including anaplastic thyroid carcinoma. Lenvatinib is is a multi-receptor tyrosine kinase inhibitor that targets multiple angiogenic and carcinogenic signaling pathways, and it has been used in the USA [14]. Age at diagnosis, tumor grade and size, extra thyroid extension, and the presence of lymph node metastases or distant metastases are all independent prognostic factors and predictors for disease-specific survival in patients with PSCCT [15].

## Conclusions

PSCCT is a rare disease that is usually diagnosed at an advanced stage and is associated with a poor prognosis. The main challenge in diagnosing PSCCT involves differentiating primary SCC arising in the thyroid from secondary SCC metastasis. The proper and accurate diagnosis of PSCCT can only be achieved through a combination of clinical, radiological, endoscopic, and immunohistologic findings. Surgery is the preferred treatment option, but it is not always curable as most cases present as locally advanced or even metastatic disease. The tumor is resistant to radiation. Aggressive combined modalities including surgery, radiation, and chemotherapy should be considered in this patient population. Molecular profiling to identify any targetable mutations should be considered as well, especially with the emergence of checkpoint inhibitors to treat such malignancies. Further studies are required to gain more insight into various aspects of this disease.
